# Chromosome-level genome assembly of *Helwingia omeiensis*: the first genome in the family Helwingiaceae

**DOI:** 10.1038/s41597-024-03568-7

**Published:** 2024-07-02

**Authors:** Yanyu Chen, Landi Feng, Hao Lin, Jianquan Liu, Quanjun Hu

**Affiliations:** Key Laboratory for Bio-Resource and Eco-Environment of Ministry of Education & Sichuan Zoige Alpine Wetland Ecosystem National Observation and Research Station, College of Life Science, Sichuan University, Chengdu, China

**Keywords:** Evolution, Plant sciences, Genome

## Abstract

*Helwingia*, a shrub of the monotypic cosmopolitan family Helwingiaceae, is distinguished by its inflorescence, in which flowers are borne on the midrib of the leaf—a trait not commonly observed in related plant families. Previous studies have investigated the development of this unusual structure using comparative anatomical methods. However, the scarcity of genomic data has hindered our understanding of the origins and evolutionary history of this uncommon trait at the molecular level. Here, we report the first high-quality genome of the family Helwingiaceae. Assembled using HiFi sequencing and Hi-C technologies, the genome of *H. omeiensis* is anchored to 19 chromosomes, with a total length of 2.75 Gb and a contig N50 length of 6.78 Mb. The BUSCO completeness score of the assembled genome was 98.2%. 53,951 genes were identified, of which 99.7% were annotated in at least one protein database. The high-quality reference genome of *H. omeiensis* provides an essential genetic resource and sheds light on the phylogeny and evolution of specific traits in the family Helwingiaceae.

## Background & Summary

Helwingiaceae is a monotypic family in the order Aquifoliales, comprising a single genus *Helwingia*. The innovative structure of this genus is that the flowers are borne on the midrib of the leaf, which is known as an “epiphyllous inflorescence”, setting them apart from other plants. In addition, the pith, leaves, and fruits of plants in this genus are traditionally used in herbal medicine to treat dysentery and as diuretic and anti-inflammatory remedies^1^. The genus includes four species, *H. chinensis*, *H. himalaica*, *H. japonica*, and *H. omeiensis*, which are all dioecious shrubs mainly found in eastern Asia^2,3^. Specifically, *H. omeiensis* is indigenous to Southwest China, and thrives in moist woodlands and on mountain slopes^2^.

Previous comparative anatomical studies suggested that changes in the position of flower primordium initiation and intercalary growth may contribute to the formation of this distinct structure^4–6^. With the development of high-throughput sequencing technologies, the genomes of three closely related species in the genus *Ilex* of the family Aquifoliaceae have been published^7,8^. However, despite the fact that RNA-seq data and the complete chloroplast genomes of three *Helwingia* species have been released^4,9,10^, a lack of genomic data remains a barrier to studying the evolutionary origin of the family.

In this study, we leveraged a combination of short reads, high-fidelity (HiFi) reads, and chromosome conformation capture (Hi-C) sequencing data to construct a chromosome-level genome assembly for *H. omeiensis*, providing the first genome resource for the family Helwingiaceae. The length of the genome assembly was 2.75 Gb, with a scaffold N50 of 127.8 Mb and a contig N50 of 6.78 Mb. We identified 1.98 Gb of repetitive elements, accounting for 72.21% of the assembled genome, as well as 53,951 protein-coding genes. The genome assembly and annotation of *H. omeiensis* will provide a critical foundation for exploring the genetic basis underpinning of this unique inflorescence structure and the phylogenetic relationships within the family Helwingiaceae.

## Methods

### Plant materials

All of the fresh materials were collected from a female adult plant of *Helwingia omeiensis* cultivated in Mount Emei Botanical Garden, Sichuan Province, China (N29°35′40, E103°22′40), and the specimens were kept at the Museum of Sichuan University. The genomic DNA was extracted from young leaves, whereas RNA was extracted from mature leaves and terminal buds.

### Library construction and sequencing

For short-read sequencing, the sample was randomly fragmented by an ultrasonic processor (Covaris S220; Woburn, MA, USA) to generate DNA fragments approximately 350 bp in length. The DNA fragments were subsequently constructed through end repair, the addition of a 3′ A tail and the ligation of adapters. Next, the library was sequenced with a DNBSEQ-G400 (BGI, Wuhan, China). The raw short reads were filtered by SOAPnuke v1.5.6^10^ to remove adapters and low-quality reads. A total of 87.36 Gb of clean data were obtained for *H. omeiensis* (Table 1).

**Table 1:** Statistics of the sequencing data of the *H.omeiensis* genome.

Library types	Molecule	Platform	Insert size	Data size (Gb)
Short-read	DNA	DNBseq	300–400 bp	87.36
HiFi	DNA	PacBio Sequel II	20 Kb	50.32
Hi-C	DNA	Illumina HiSeq X Ten	—	221.52
RNA-Seq	RNA	Illumina HiSeq X Ten	—	10.46

For HiFi (high-fidelity) sequencing, high-quality genomic DNA was sheared using Megaruptor^®^ 3 (Diagenode), and subreads with a length of 20 kb were further selected using Sage ELF to prepare the PacBio HiFi libraries in CCS mode on the Pacific Biosciences Sequel II System (Supplementary Figure S1). Finally, 50.32 Gb of long clean reads were generated (Table 1), with mean lengths of 13.0 kb and 14.5 kb, respectively.

Hi-C technology captures sequence interactions between all DNA segments within chromosomes to obtain information on interactions between segments of the genome for assisted genome assembly^11^. Fresh leaves of the same individual were used to construct Hi-C libraries, and the MboI restriction enzyme was used for DNA ligation. After tailing, pulldown, and adapter ligation, the DNA library was sequenced on an Illumina HiSeq X Ten System (BGI, Wuhan, China) with a strategy of 2 × 150 bp. After filtering low-quality reads, 221.52 Gb of clean Hi-C data were obtained (Table 1).

### RNA sequencing

Mature leaves and young terminal buds of the same individual were collected for RNA extraction. The RNA-seq library was constructed using the Illumina standard protocol (San Diego, CA, United States) and sequenced on the Illumina HiSeq X Ten platform (BGI, Wuhan, China). The raw data were filtered by Cutadapt v1.16^12^ to remove adapters and low-quality reads. After quality control by FastQC v0.11.8 (https://github.com/s-andrews/FastQC), 10.46 Gb of paired-end short clean reads were generated from the RNA-seq library (Table 1).

### Genome survey and *de novo* assembly

**Fig. 1:**
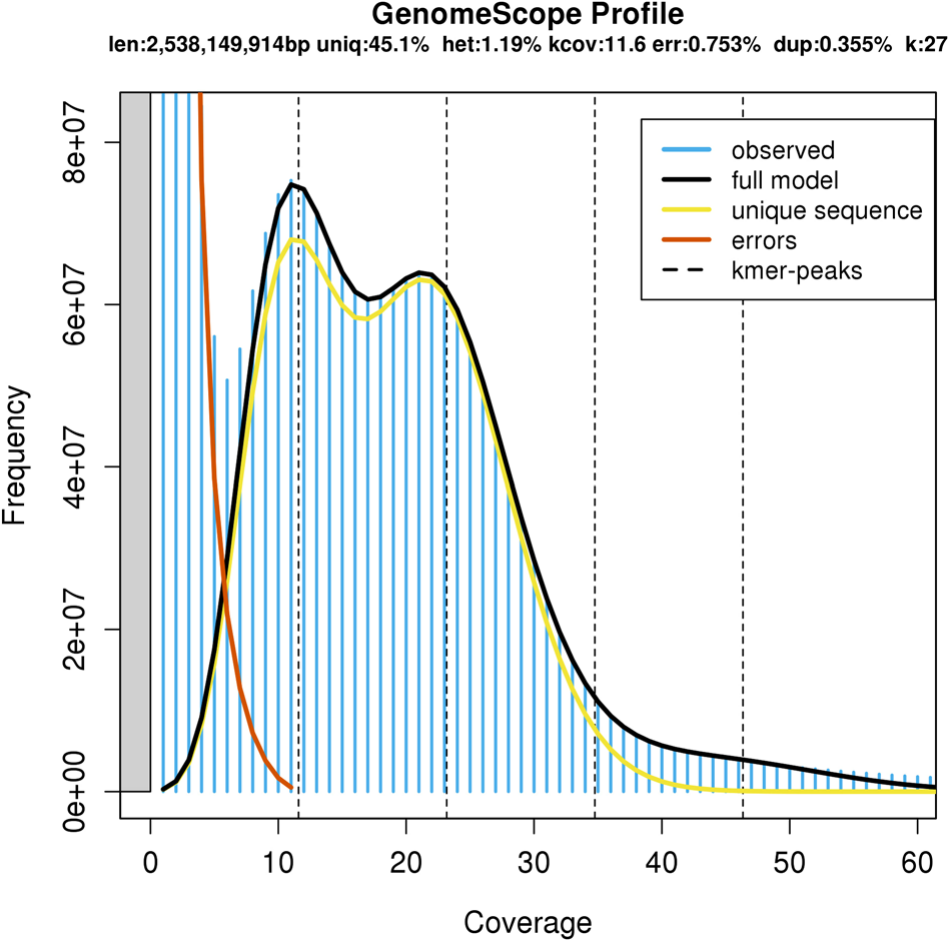
Distribution profiles of 27-mer analysis of short reads.

**Fig. 2:**
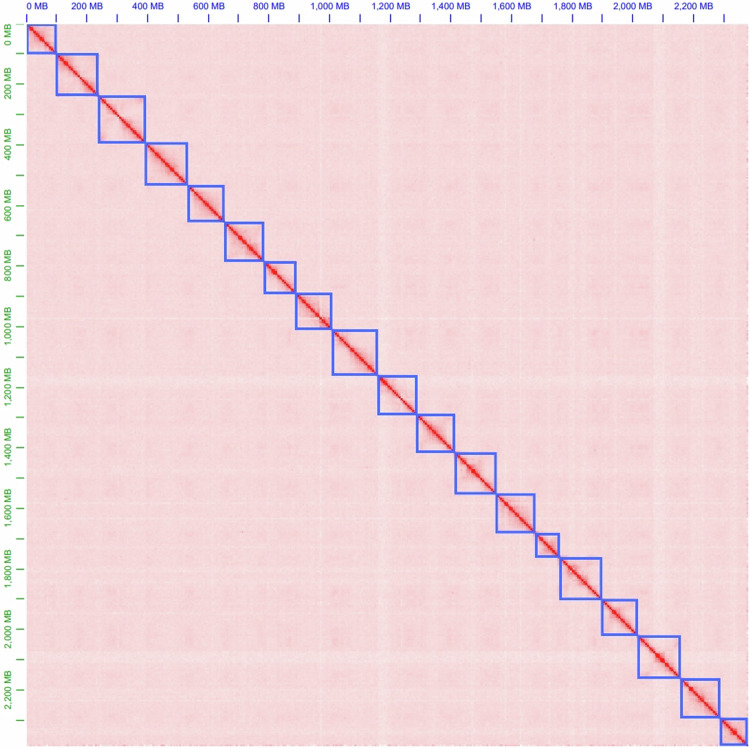
The Hi-C interactive heatmap of 19 pseudo-chromosomes of *H. omeiensis*.

**Fig. 3:**
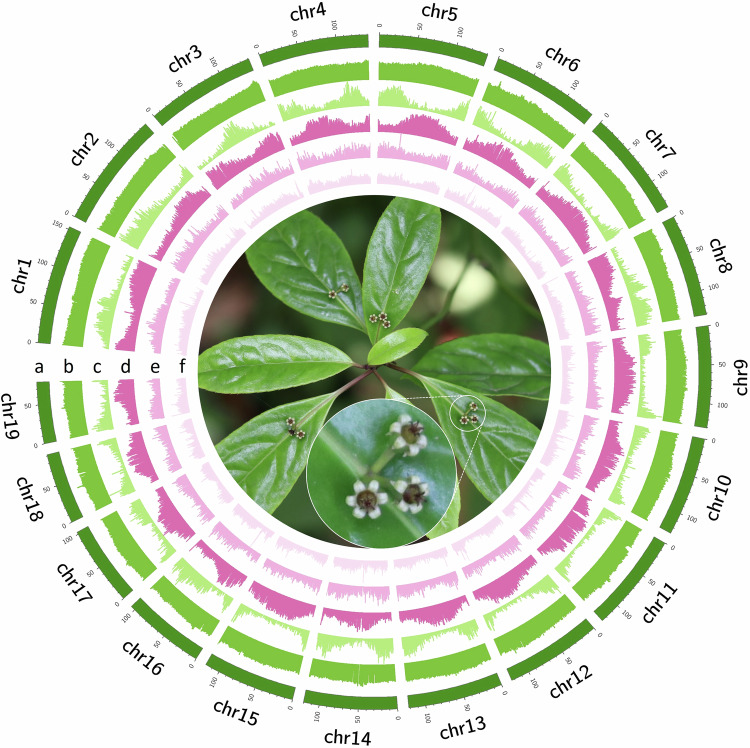
Circos plot of genomic characteristics and annotation of the *H. omeiensis* genome assembly (1 Mb window size). The data from the outer to the inner circles are as follows: (**a**) length of 19 pseudochromosomes, (**b**) GC density, (**c**) gene density, (**d**) *Gypsy* retrotransposon density, (**e**) *Copia* retrotransposon density, and (**f**) DNA transposon density.

Jellyfish v2.1.4^13^ was used to quickly count *K*-mer frequencies ranging from 17 to 31, and then GenomeScope^14^ predicted genomic features using a *K*-mer-based statistical approach (Supplementary Table S1). The *H. omeiensis* genome was estimated to be 2.54 Gb in size, with a heterozygosity rate of 1.19% and repetitive sequences accounting for 54.85% of the total length of the genome (Fig. 1). Using 50.32 Gb of clean HiFi reads with hifiasm v0.19.6-r595^15^, we generated a genome assembly of 2.92 Gb in size with a contig N50 of 6.21 Mb. Following that, Chromap v0.2.5-r473^16^ was utilized to align Hi-C clean reads to the contig assembly, and according to the strength of interactions between pairs of reciprocal sequences, YaHS v1.2a.1^17^ was used to anchor contigs onto 1,584 scaffolds. Next, using Juicebox v1.11.08^18^, we visualized the Hi-C contact maps of the scaffold assembly and made final refinements to the genome assembly. With reference to chromosome counts indexed in the Chromosome Counts Database (CCDB)^19^ (https://ccdb.tau.ac.il/) and the whole-genome Hi-C interaction heatmap, we identified the 19 longest scaffolds as pseudo-chromosomes (Fig. 2). TGS-GapCloser v1.2.1^20^ filled 75 of the 1,011 gaps in the scaffold assembly based on HiFi reads. The final assembly had a total length of 2.75 Gb, with a contig N50 of 6.78 Mb. The length of 19 pseudochromosomes was 2.38 Gb, with a maximum chromosome length of 153.79 Mb (Table 2). Since there is no reference genome for this species, we numbered the chromosomes in order from largest to smallest (Fig. 3 and Table 3).

**Table 2:** Statistics of chromoslme-level genome assembly of *H. omeiensis*.

Features	Statistics
Assembly size (bp)	2,748,316,618
Largest sequence length(bp)	153,486,838
Counts of scaffold sequences	1,584
Scaffold N50 (bp)	127,781,096
Counts of scaffold N50	10
Scaffold N90 (bp)	16,574,032
scaffold L90	23
Contig N50 (bp)	6,783,361
Contig L50	119
Contig N90 (bp)	927,000
Counts of contig N90	502
GC content(%)	35.19
N Length	193,200
N content (%)	0.007
BUSCO completeness (%)	98.2

**Table 3:** Statistics of 19 chromosomes of *H. omeiensis* genome assembly.

No.	Length (bp)	Number of gaps	GC percentage (%)
chr1	153486838	37	35.66
chr2	149133724	45	34.64
chr3	141481271	21	35.36
chr4	141123649	33	35.04
chr5	140777598	40	35.28
chr6	139309107	48	35.52
chr7	138197217	25	35.42
chr8	131542532	50	35.34
chr9	130545343	39	35.49
chr10	127781096	37	35.20
chr11	126849556	36	35.61
chr12	123901092	28	35.34
chr13	121841527	30	35.29
chr14	120348231	32	34.64
chr15	119169170	26	35.52
chr16	105907320	29	34.74
chr17	102947481	24	35.28
chr18	91427918	18	35.36
chr19	79123487	20	35.16

### Gene annotation

To perform a comprehensive prediction of protein-coding genes, the GETA v2.5.6 pipeline (https://github.com/chenlianfu/geta) was used for automatic genome-wide annotation. First, RepeatModeler v2.0.3^21^ and DeepTE^22^ were used for self-training and to construct a repeat library. On this basis, RepeatMasker v4.1.2-p1^23^ was employed to predict and combine repetitive elements for homology-based methods. The analysis revealed that 72.21% of the genome was composed of repetitive sequences, including 46.39% long-terminal repeat (LTR) retrotransposons and 19.43% DNA transposons (Table 4).

**Table 4:** Classification of repetitive sequences of *H. omeiensis* genome.

Type			Number of elements	Sequence length (bp)	Percentage (%)
Retroelements	LTR elements	Gypsy	701,531	1,006,106,967	36.60
		Copia	185,233	172,265,891	6.26
		Caulimovirus	12,359	13,943,858	0.50
		Cassandra	410	573,724	0.02
		DIRS	128	78,353	0.00
		Total	929,626	1,275,113,390	46.39
	LINE		26,024	43,464,843	1.58
	SINE		13,225	2,251,476	0.08
	Total		968,875	1,320,734,580	48.05
DNA transposons			1,318,664	534,237,277	19.43
Rolling-circles			6,346	12,191,657	0.44
Simple repeat			366,173	17,496,450	0.63
Low complexity			62,060	4,145,299	0.15
Satellite			13,489	11,312,672	0.41
snRNA			12	23,995	0.00
tRNA			43	40,922	0.00
rRNA			1,244	4,406,540	0.16
Unknown			441,975	132,712,782	4.82
Total			3,178,881	1,984,642,021	72.21

After masking repetitive sequences in the genome, three strategies (homology-based, RNA-seq-guided, and *ab initio* methods) were used for the annotation process. For the RNA-seq-guided method, the RNA sequencing data were provided to HISAT2 v2.1.0^24^ and SAMtools v1.11^25^ to map the data to the repeat-masked genome. Then, TransDecoder v5.5.0 (https://github.com/TransDecoder/TransDecoder) was used to predict the open reading frame (ORF), and filter out the gene models with identities greater than 80% at the amino acid level between pairs to obtain nonredundant results. Protein sequences from *Vitis vinifera*, *Arabidopsis thaliana*, *Solanum lycopersicum*, *Daucus carota*, and *Ilex latifolia* were aligned to the query genome as homologous proteins using GeneWise v2.4.1^26^ to estimate protein-coding genes (Supplementary Table S2). *Ab initio* prediction was carried out with AUGUSTUS v3.4.0^27^, which guided by previous prediction results. Based on the GETA pipeline, all the outputs were validated using HMMER v3.3.2^28^ and NCBI-BLAST + v2.13.0 + before being integrated into a complete and nonredundant set of gene annotations.

Following the alignments by DIAMOND v2.0.15^29^, gene functions were indicated using the Nonredundant Protein Sequence Database (NR)^30^, InterPro^31^, UniProt^32^, and EggNOG^33^ with an e-value of 1e-5. In addition, GO annotation was performed by KOBAS^34^ (http://kobas.cbi.pku.edu.cnwas) aligned with the *Arabidopsis thaliana* database.

## Data Records

All the raw sequencing reads of *H. omeiensis* were uploaded to the NCBI database under accession number SRP435213^35^. The genome assembly had been submitted to Genome Warehouse in China National Center for Bioinformation under accession number GWHEQHK00000000^36^ and European Nucleotide Archive (ENA) with accession number GCA_964187755.2^37^. The annotation files of the genome are available in the figshare database: 10.6084/m9.figshare.22817414.v3^38^.

## Technical Validation

### Evaluation of the genome assembly and annotation

To assess the integrity of the assembly, short reads were mapped to the genomes using minimap2^39^, giving a mapping rate of 96.59% and a genome coverage of 99.85%. The alignment rate of RNA sequencing reads was 96.95% and 94.10% for two *H. omeiensis* samples by HISAT2 v2.1.0 (Supplementary Table S3)^24^. The completeness and accuracy of the final genome assembly were checked by Benchmarking Universal Single-Copy Orthologs (BUSCO) v5.4.2^40^ with eudicots_odb10. The results showed that 98.2% of orthologs of eudicots could be identified in the assembly (Supplementary Figure S2). Moreover, the values evaluated by Merqury v1.3^41^ based on short reads also showed high consensus quality (accuracy > 99.99%, QV > 58) and low base-level error rates (1.37 × 10^−6^). In addition, the LTR Assembly Index (LAI) score of the whole-genome assembly was calculated to be 24.52, exceeding that of rice (MSUV7) and *Arabidopsis* (TAIR10), reaching the ‘gold quality’^42^. These results demonstrated that the assembly is reliable and has high base-level accuracy, high completeness, and high contiguity.

**Fig. 4:**
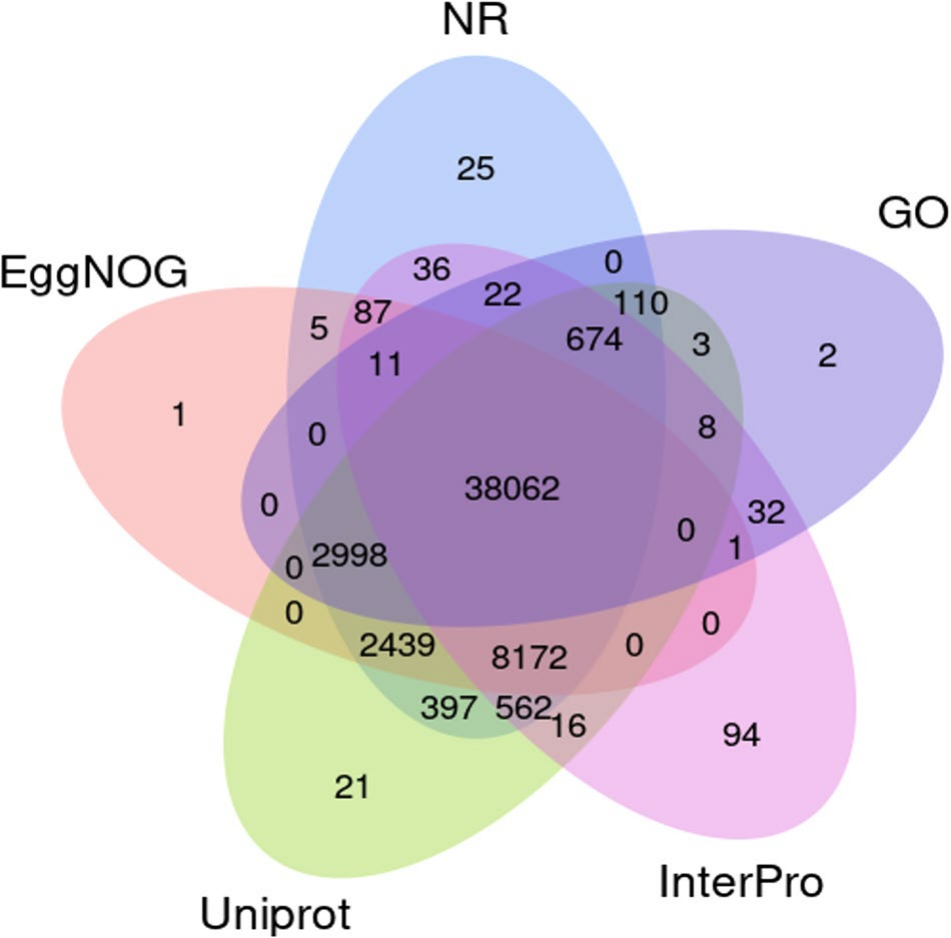
Venn diagram displaying the matches of genes of *H. omeiensis* in five public protein databases.

Via multiple annotation approaches, we identified 53,951 protein-coding genes in the *H. omeiensis* genome (Table 5). BUSCO analysis showed the completeness of predicted genes was 94.5% (Supplementary Figure S2). The functional analysis revealed that 99.7% of the protein-encoding genes could be annotated in at least one of five public databases (Fig. 4).

**Table 5:** Predicted protein-coding genes of *H. omeiensis*.

Features	Number	Size mean (bp)	% of the genome
gene	53,951	5,145.9	10.1
exon	208,603	341.6	2.6
intron	154,652	1,334.4	7.5
mean exons per gene	3.9	—	—
mean introns per gene	2.9	—	—
five_prime_utr	25,149	171.8	0.2
three_prime_utr	16,528	282.9	0.2

### Supplementary information

Supplementary Information

## Data Availability

(1) SOAPnuke v1.5.6: parameters: -n 0.01 -l 20 -q 0.1 -i -Q 2 -G -M 2 -A 0.5 -d (2) Cutadapt v1.16: parameters: -a AGATCGGAAG -q 20 All the other software and pipelines not listed or described in the methods section used the default parameters.
