# Supplementary material for: Chromosome-level genome assembly of Helwingia omeiensis: the first genome in the family Helwingiaceae
Source: Sci Data. 2024 Jul 2;11:719. doi: 10.1038/s41597-024-03568-7 (PMC11220072; doi:10.1038/s41597-024-03568-7)
Supplement: Supplementary file 1 — Supplementary Information [file 41597_2024_3568_MOESM1_ESM.pdf]

Supplementary Information

Figure S1. Distribution of subreads length. 1

Figure S2. BUSCO assessment results of the genome and predicted genes of *H. omeiensis*. .. 1

Table S1. Statistics of genome survey based on different *K*-mer analysis 2

Table S2. Summary of protein sequences used in homology-based gene prediction 2

Table S3. Numbers of RNA sequencing reads mapped to *H. omeiensis* genome..................... 2

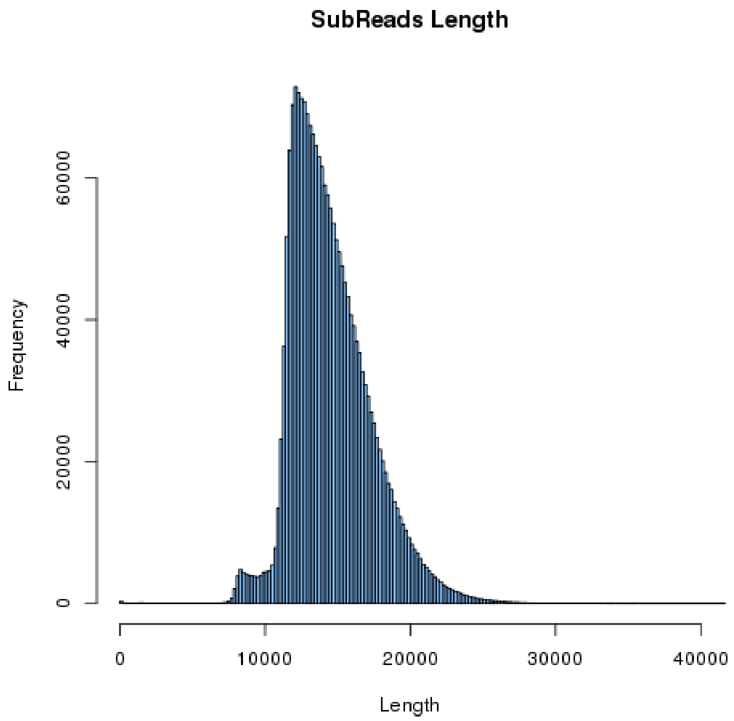

Figure S1. Distribution of subreads length.

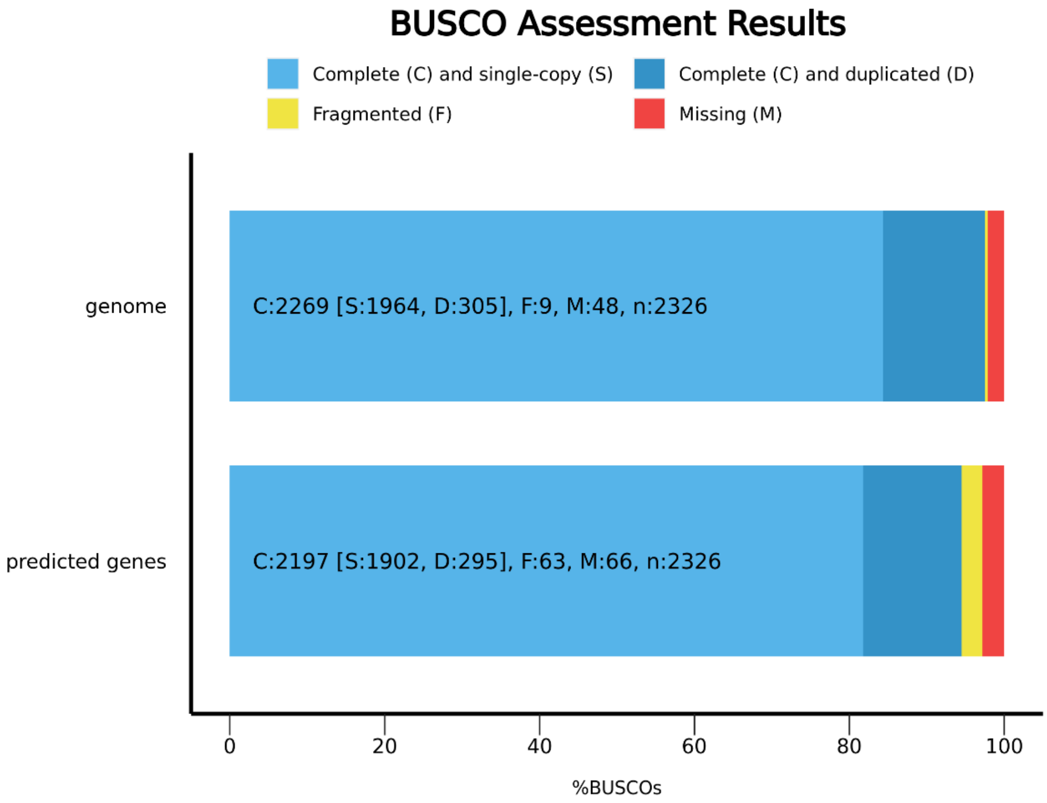

Figure S2. BUSCO assessment results of the genome and predicted genes of *H. omeiensis*.

Table S1. Statistics of genome survey based on different *K*-mer analysis

***K*-mer**	**Used base**	**Heterozygos Rate (%)**	**Genome Size (Mb)**	**Repeat Rate (%)**	**Err Rate (%)**	**Depth**
17	86,894,682,711	1.27	2,493.29	81.34	0.53	34.85
19	86,742,373,334	1.29	2,557.31	71.17	0.70	33.92
21	86,702,324,379	1.28	2,558.01	65.34	0.75	33.89
23	86,693,647,367	1.25	2,551.78	61.24	0.76	33.97
25	86,694,394,256	1.22	2,545.90	57.87	0.76	34.05
27	86,697,967,091	1.19	2,538.15	54.85	0.75	34.16
29	86,703,488,826	1.15	2,531.27	52.15	0.75	34.25
31	86,710,743,692	1.13	2,526.30	49.75	0.74	34.32

Table S2. Summary of protein sequences used in homology-based gene prediction

**Species**	**Family**	**Data source**	**Accession number**
Arabidopsis thaliana	Brassicaceae	NCBI	GCF_000001735.3
Vitis vinifera	Vitaceae	Ensembl	PN40024.v4
Solanum lycopersicum	Solanaceae	NCBI	GCF_000188115.5
Daucus carota	Apiaceae	NCBI	GCF_001625215.1
Ilex latifolia	Aquifoliaceae	NGDC	GWHBIST00000000

Table S3. Numbers of RNA sequencing reads mapped to *H. omeiensis* genome.

Sample	Total pairs	Pair end mapped reads	Pair end alignment rate (%)	Single end mapped reads	Single end mapped alignment rate (%)	Overall alignment rate (%)
Mature leaf	20,780,741	19,411,769	93.42	957,463	4.61	96.95
Young terminal bud	20,474,547	18,504,209	90.37	1,042,739	5.09	94.10
